# Effective Use of Linear DNA in Cell-Free Expression Systems

**DOI:** 10.3389/fbioe.2021.715328

**Published:** 2021-07-20

**Authors:** Megan A. McSweeney, Mark P. Styczynski

**Affiliations:** Georgia Institute of Technology, School of Chemical & Biomolecular Engineering, Atlanta, GA, United States

**Keywords:** cell-free expression, linear expression template, nuclease inhibition, genetic circuits, rapid prototyping, DNA aptamers

## Abstract

Cell-free expression systems (CFEs) are cutting-edge research tools used in the investigation of biological phenomena and the engineering of novel biotechnologies. While CFEs have many benefits over *in vivo* protein synthesis, one particularly significant advantage is that CFEs allow for gene expression from both plasmid DNA and linear expression templates (LETs). This is an important and impactful advantage because functional LETs can be efficiently synthesized *in vitro* in a few hours without transformation and cloning, thus expediting genetic circuit prototyping and allowing expression of toxic genes that would be difficult to clone through standard approaches. However, native nucleases present in the crude bacterial lysate (the basis for the most affordable form of CFEs) quickly degrade LETs and limit expression yield. Motivated by the significant benefits of using LETs in lieu of plasmid templates, numerous methods to enhance their stability in lysate-based CFEs have been developed. This review describes approaches to LET stabilization used in CFEs, summarizes the advancements that have come from using LETs with these methods, and identifies future applications and development goals that are likely to be impactful to the field. Collectively, continued improvement of LET-based expression and other linear DNA tools in CFEs will help drive scientific discovery and enable a wide range of applications, from diagnostics to synthetic biology research tools.

## Introduction

Cell-free expression systems (CFEs) are powerful tools for the execution of arbitrary genetic programs or the synthesis of proteins *in vitro*. One of the most common and affordable forms of CFE, the lysate-based system, is composed of a crude cellular extract (typically from *E. coli*, but lysates from other organisms are useful for specific applications) combined with supplemented cofactors and substrates essential for transcription and translation. CFEs offer several advantages over the use of whole-cell *in vivo* systems (Khambhati et al., 2019). Transport limitations inherent to whole-cell systems due to the cell membrane are reduced in CFEs because they have no membrane, yielding improved control over plasmid dosage, pH, and inducer levels (Swartz 2006). CFEs also eliminate cellular toxicity issues that sometimes arise *in vivo* from the expression of certain proteins, with the additional effect of avoiding plasmid instability often caused by toxicity (Katzen et al., 2005). CFEs have expedited research on key biological principles (Nirenberg and Leder 1964) and have been applied in contexts ranging from industrially relevant large-scale protein production (Zawada et al., 2011) to upstream screening for glycoprotein synthesis (Schoborg et al., 2018). CFEs are particularly attractive for sensor development, with applications ranging from environmental sensors (Verosloff et al., 2019; Thavarajah et al., 2020) to user-friendly biomedical diagnostics (McNerney et al., 2019).

**FIGURE 1:**
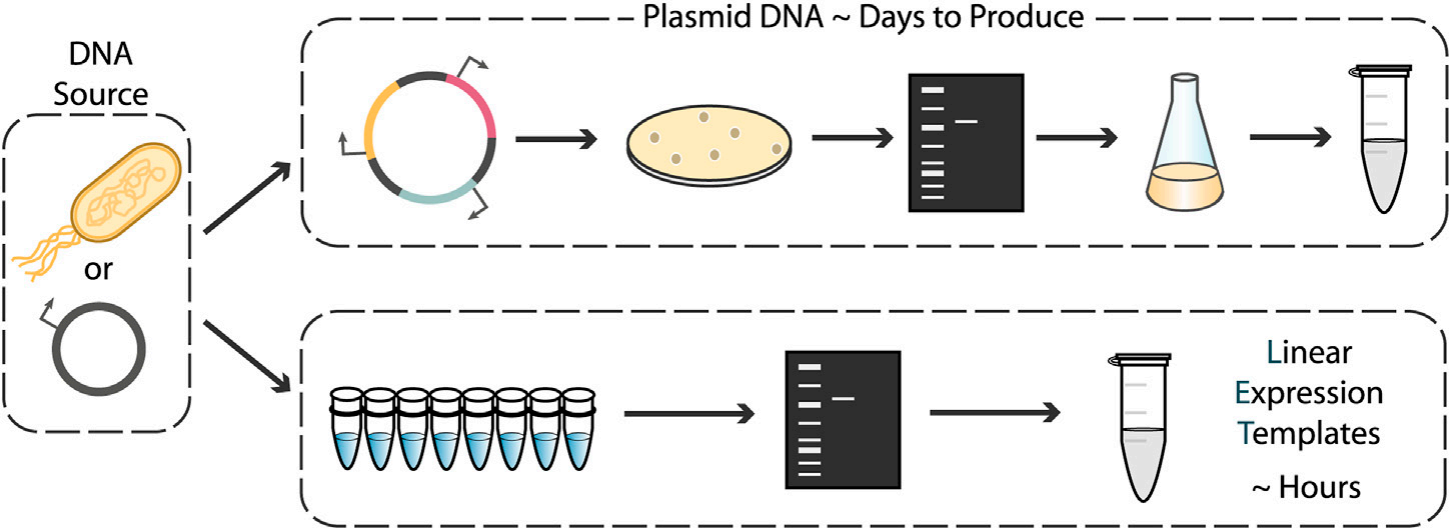
Strategies for preparing DNA templates for use in cell-free expression systems. Traditional plasmid cloning protocols involving construct assembly, transformation, screening, and plasmid purification take days to complete. Linear expression templates can be made via PCR from genomic DNA or plasmid templates and used directly after amplicon verification and purification, drastically reducing DNA template preparation time.

The configuration of CFEs as a membrane-less solution of expression machinery, compared to the membrane-compartmentalized individual cells of *in vivo* systems, not only reduces the transport limitations that would have been imposed by the membrane, but also makes the use of linear expression templates (LETs) a viable option for CFEs. For *in vivo* systems, the use of plasmids is required for stable expression without genomic integration. Since delivery of DNA into cells happens with low efficiency, successfully transformed cells must be selected for and then expanded, meaning that the DNA vector must replicate during cell growth to avoid growth-associated dilution and loss. Plasmid construction requires cloning, *in vivo* synthesis, and plasmid isolation, a process that takes days for each new construct. However, LETs—which typically consist of a promoter region, gene coding sequence, and transcriptional terminator—can be quickly and easily produced *in vitro* via polymerase chain reaction (PCR) from existing plasmid DNA or genomic DNA (Figure 1). With techniques such as Golden Gate assembly, multiple LETs can be rapidly assembled into complex constructs entirely *in vitro*. Using PCR products as expression templates rather than plasmids can decrease the “primers-to-testable-DNA” time from days to only a few hours. This can facilitate high-throughput screening and significantly accelerate the prototyping cycle time of multicomponent genetic circuits to a standard business day (Sun et al., 2013).

Additionally, LETs allow for expression of toxic genes in CFEs that otherwise would be difficult to clone into a plasmid. The LET construct for the toxic gene can be amplified directly from genomic DNA or from a plasmid without a promoter upstream of the gene. Without a promoter, the plasmid containing the toxic gene can be cloned because the gene will not be expressed *in vivo* as normal. This can then be used as a template for a LET, with the promoter sequence added via primers during PCR.

Despite the numerous benefits of LETs, plasmids remain the most widely used DNA template in CFEs due to their resistance to degradation. DNA nucleases native to *E. coli* are present in the crude cellular lysate and remain after lysate purification. These nucleases readily digest linear double-stranded (ds) or single-stranded (ss) DNA fragments in the reaction to an extent not observed for circular plasmids, causing LETs to have a much shorter half-life than plasmids. This leads to much lower protein yields or diminished function of genetic circuits, which has in turn slowed adoption of LETs for CFEs.

However, there exist several approaches for stabilizing linear DNA that lessen the impacts of these phenomena and allow for the effective use of LETs in crude lysate-based CFEs. In this review, we will describe these advances and compare their effectiveness and limitations. Then, we will summarize select applications where LETs have been used to expedite circuit prototyping cycles, rapidly screen synthetic regulators, express toxic proteins, and more. Lastly, we will discuss the restrictions of the existing nuclease inhibition strategies, recognize areas with critical need for improvement, and identify future development goals.

## Approaches for Stabilizing Linear DNA in Cell-Free Expression Systems

While crude bacterial lysate is widely used for CFEs, purified recombinant proteins can also constitute the basis for CFEs. PURExpress (NEB), PUREfrex 2.0, and Magic PURE are three commercially available recombinant systems based on the original PURE system developed by Shimizu et al. (2001) (See also Laohakunakom et al. (2020)). Purchasing these systems from commercial vendors saves valuable laboratory time compared to typical modern protocols for in-house preparation of crude cell extract that typically take three days and over 10 h of active labor (Kwon and Jewett 2015). Perhaps more importantly, these systems have minimal nuclease and protease activity compared to crude lysates, making expression from LETs much easier than in lysates.

However, crude lysate-based CFEs still dominate the field based in part on their affordability. For example, the PURExpress *in vitro* protein synthesis kit costs $0.35-$0.65 per 1 uL reaction while a crude lysate-based system costs $0.02-$0.04 per 1 uL reaction (Pardee et al., 2014). Recently, robust methods have been reported for in-house production of recombinant protein CFEs by coculturing and inducing expression of all 36 proteins present in the commercial systems (Lavickova and Maerkl 2019). This “OnePot” method can achieve high protein yields at a cost closer to $0.09 per μL. While this is significantly lower than the cost of the PURExpress system, it is still more expensive than crude lysate CFEs and laborious in its own way; it remains to be seen whether this approach will be adopted widely.

**FIGURE 2:**
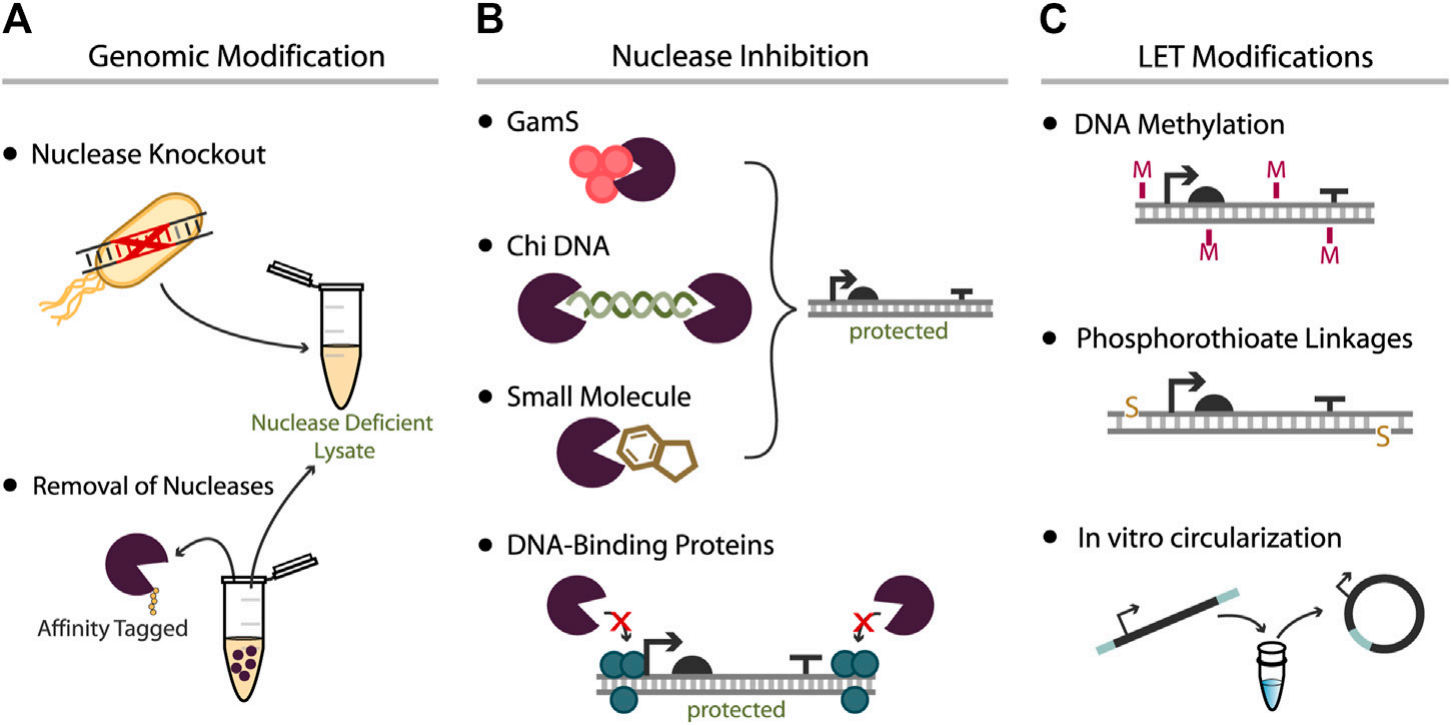
Approaches used to increase expression yield from LETs. **(A)** Bacteria can be genetically modified prior to lysate extraction either by deletion of nuclease genes or by the fusion of affinity peptides to nuclease genes for later removal during lysate processing. Both methods aim to produce a lysate with negligible nuclease activity. **(B)** Nuclease inhibitors can be added to cell-free reactions to mitigate activity of specific nucleases. This effect can be achieved with direct RecBCD inhibitors such as GamS, Chi DNA, and certain small molecules, or via the addition of DNA-binding proteins that interact with the LETs. **(C)** The LET can also be modified to better protect the construct from nuclease degradation: methylation of PCR-generated LETs can mimic the chemistry of native DNA, phosphorothioate linkages can be added to the ends of LETs via modified primers, or amplicons with appropriately designed primers can be recircularized prior to CFE.

The financial benefits of lysate-based CFEs, among other advantages, have motivated substantial effort toward addressing one of its major shortcomings: the effects of nucleases in crude extracts. Significant linear DNA degradation is attributed to exonuclease V, the product of the *recBCD* operon. As a result, previous efforts have attempted to remove, inhibit, or deter RecBCD activity on LETs (Figure 2), with varying success. The relative effectiveness of each approach is listed in Table 1. The most common approaches to achieve these goals include genomic removal of nucleases, nuclease inhibition, and protective linear DNA modifications.

**TABLE 1:** Effectiveness of different nuclease inhibition strategies quantified by their ability to improve LET-based expression in *E. coli* CFEs. Values with an asterisk were inferred from figures in the corresponding reference.

LET stabilization approach		Improvement	Metric	References
**Genomic modifications**	Δ*recCBD::P*_*lac*_ *-red-kan-*Δ*endA*	3–6x	Fold change from WT strain	Michel-Reydellet et al. (2005)
	Affinity tag removal of RecD and PNPase	4x	Fold change from WT strain	Seki et al. (2009)
**Nuclease inhibition**	GamS	37.6%	Percentage of plasmid expression	Sun et al. (2013)
	Chi DNA	23%*	Percentage of plasmid expression	Marshall et al. (2017)
	Small molecule RecBCD inhibitors	250%* (CID 697851) 300%* (CID 1517823)	Percent increase from no inhibitor	Shrestha et al. (2014)
	ssCro	23%	Percentage of plasmid expression	Zhu et al. (2020)
	Ku	8%*	Percentage of plasmid expression	Yim et al. (2020)
**LET modifications and enhanced design**	DNA methylation	32% (*dam* methyltransferase) −18% (CpG methyltransferase)	Percent increase from unmethylated LET	Zhu et al. (2020)
	Terminal phosphorothioate (PT) linkages (x2)	36%*	Percent increase from unmodified LET	Sun et al. (2013)
	3′-tail mRNA secondary structures	92%* (poly(G) tail) 265%* (T7 terminator)	Percent increase from LETs lacking 3′ secondary structures	Ahn et al. (2005)

### Genomic Modifications

In *E. coli*, endonucleases and exonucleases are critical for proper cell function and are essential enzymes involved in double-stranded break repair and recombination events. The predominant endonuclease in *E. coli* is endonuclease I, encoded by the *endA* gene. The dominant source of exonuclease activity in *E. coli* comes from exonuclease V, the product of the *recBCD* operon (Kuzminov and Stahl 1997). In this complex, RecB has both helicase and nuclease activity, and RecD serves only as a helicase. Attempts have been made to engineer *E. coli* mutants that lack the activity of one or several of these subunits (Figure 2A). For example, a cell extract made from an A19Δ*recD*Δ*endA* mutant was used for cell-free protein synthesis and tested with LETs for nuclease activity (Michel-Reydellet et al., 2005). Plasmid-based expression was slightly lower in the mutant extract compared to the wild type, suggesting that the *endA* deletion did not improve plasmid stability. However, extracts from both strains possessed significant linear DNA degradation as evidenced by low protein yields from the LETs. And while these results showed almost equal expression from plasmids between strains, others have reported that extracts of exonuclease-deficient strains exhibit significant loss in translational activity (Ahn et al., 2005).

Knockout strains that lack the entire *recBCD* operon have also been explored. Complete *recBCD* knockouts create strains that are extremely slow-growing, cannot support recombination, and do not allow for replication of many plasmids (Yu et al., 2000). To address this, *recBCD* can be replaced with the Red recombination system from bacteriophage λ to remove exonuclease activity while allowing recombination and adequate growth rates (Murphy 1991; Michel-Reydellet et al., 2005). Lysates made from this mutant yielded over three times more protein from PCR-generated LETs compared to the wild-type extract.

Since the growth defects of these knockout strains make the culture steps in lysate preparation more time-consuming, removing nucleases from the lysate post-harvest has been explored as an alternative option. Insertion of streptavidin-binding peptide tag sequences in the 3’ termini of RecD and the gene encoding polynucleotide phosphorylase (PNPase) was shown not to effect *E. coli* growth (Seki et al., 2009). Lysates made from these modified strains were treated with an affinity purification resin to remove tagged proteins, leading to approximately four times as much GFP production from LETs compared to wild-type BL21 (Seki et al., 2009). Expanded to multiple other nucleases simultaneously, this approach could potentially have a substantial impact on linear DNA stability. That being said, genes that overlap on the chromosome cannot be tagged (and thus, removed), and physical limitations of the resin such as its binding capacity and specificity can limit the success of this approach. Additionally, the resin-treated lysate with the greatest improvement in LET protein yield exhibited a 15% loss in plasmid-driven protein expression, which reduces the overall potential yield of the system.

### Nuclease Inhibition

Inhibiting nucleases present in the lysate instead of removing them completely is another promising approach to improving expression from LETs (Figure 2B). This strategy is potentially simpler and more easily generalizable than genetic modifications, since the addition of supplements to CFEs can be applied to lysates of any strain background. Multiple strategies for nuclease inhibition have been reported.

One widely used method for nuclease inhibition is inclusion of the bacteriophage λ protein GamS in CFEs. GamS is an inhibitor of RecBCD that can protect linear DNA from degradation *in vivo* and in crude extracts (Murphy 1991; Sitaraman et al., 2004). GamS has been used in CFEs to allow expression from LETs containing various promoters, including T7, natural and synthetic σ^70^, and well-characterized inducible promoters (Sun et al., 2013). With GamS supplementation, LETs with a strong σ^70^ promoter yielded 37.6% of that from a plasmid, a large improvement from the 2% LET yield in the absence of GamS. The GamS protein can be purified with affinity tagging or purchased commercially specifically for use in CFEs. Enhanced extracts can also be made from strains that express GamS to improve LET performance without the need to exogenously add protein. These GamS-containing extracts doubled LET protein yield compared to extracts without GamS (Contreras-Llano et al., 2020). While this method is more cost-effective and avoids any negative effects caused by the protein storage buffer, the observed benefits in LET performance are far less than those seen when purified GamS is added. Additionally, enriched lysates made from plasmid-carrying strains can suffer from overall decreases in efficiency, a consequence of the burdens of plasmid maintenance (Zawada and Swartz 2006).

The use of Chi DNA for RecBCD inhibition also avoids the costly purchase or reagent-intensive purification of proteins. Chi DNA are dsDNA oligos containing the short DNA motif (5′-GCTGGTGG-3′) referred to as a Chi (crossover hotspot instigator) site. These sites can be found throughout the genome of *E. coli* and are known to be involved in recombination events. Chi sites act as recognition sequences for RecBCD and other proteins involved in recombination (Smith et al., 1981). Addition of dsDNA composed of six repeated Chi sequences (with spacers) to the cell-free reaction has been shown to stabilize LETs and improve protein yield from undetectable levels to approximately 23% of that from a plasmid (Marshall et al., 2017).

RecBCD activity can also be inhibited by organic small molecules. From an *in vivo* screen of 326,100 organic small molecules, several compounds were identified to inhibit the nuclease activity of RecBCD and AddAB, a RecBCD analog native to *B. subtilis* and other bacteria (Amundsen et al., 2012). Many of these compounds showed reduced helicase and Chi-cutting activity from RecBCD as well. Two of these small molecules, CID 697851 and CID 1517823, improved expression from LETs in CFEs as much as 200% at some concentrations compared to reactions with no RecBCD inhibitors (Shrestha et al., 2014). However, expression remained far less than that from a plasmid.

GamS, Chi DNA, and small molecule RecBCD inhibitors all function by binding directly to RecBCD, but nuclease inhibitors can also bind directly to the LET to protect it from degradation. For example, adding single-chain Cro (ssCro) to a cell-free reaction protects LETs containing the ssCro operator recognition sequences from RecBCD degradation (Jana et al., 1998; Zhu et al., 2020). These ssCro operators can be easily added to LETs through PCR. Addition of ssCro improved LET expression over 6-fold, resulting in about 23% of plasmid-based expression (Zhu et al., 2020). DNA-binding proteins can also inhibit degradation from other exonucleases. Ku, for example, is a DNA-binding protein with homologs found in eukaryotes and prokaryotes that aids in nonhomologous end joining (Aravind and Koonin 2001). Ku binds directly to dsDNA termini and is proven to reduce degradation by AdnAB, a helicase/nuclease in mycobacteria (Sinha et al., 2009). Ku has been used recently in *E. coli* CFEs to protect LETs and improved transcription significantly, but those yields were only about a third of those transcribed when using GamS or Chi DNA oligos as inhibitors (Yim et al., 2020). However, because of Ku’s different mechanism for LET protection, it was proven to be the most effective in lysates made from diverse bacteria. In *B. subtilis* and *C. glutamicum* extracts, Ku-protected LETs improved transcription 4.43- and 1.58-fold, respectively, compared to the no inhibitor control (Yim et al., 2020). This is notable improvement, considering GamS and Chi DNA addition to *C. glutamicum* CFEs actually reduced RNA yield slightly. While Chi DNA enhanced transcription from LETs in *B. subtilis* CFEs, Ku was about twice as effective.

### Linear Expression Template Modifications and Enhanced Design

Chemical modification of LETs can also reduce their enzymatic degradation in CFEs (Figure 2C). In *E. coli*, methylation of genomic DNA provides some protection against restriction endonucleases (Marinus and Løbner-Olesen 2014). PCR-generated LETs, by virtue of being synthesized *in vitro*, are unmethylated. Post-synthesis methylation via commercially available enzymes was thus tested as an avenue for extra protection against degradation of LETs. LET methylation via *dam* methyltransferase (adenosine-specific) increased protein yield by 32% while methylation by CpG methyltransferase (cytosine-specific) actually lowered protein yield by 18% (Zhu et al., 2020). This decrease could be because CpG methyltransferase is a eukaryotic enzyme, yielding methylation patterns that might still appear foreign in *E. coli*. This hypothesis would suggest that it’s not simply the presence of methyl groups added by *dam* methyltransferase that led to increased yield, but the prokaryotic methylation pattern on the LET disguising it as native DNA. The potential number of methyl groups that can be incorporated throughout the LET is restricted with this method, since the commercial methyltransferases act only at sequence-specific sites. These sites can easily be added at regions flanking the gene, but the concentration of methylation sites within the gene itself will be fixed.

Noncanonical DNA backbones and nucleotides can also be used to decrease LET susceptibility to degradation by lysate nucleases. Phosphorothioate (PT) linkages have been shown to restrict exonuclease digestion *in vivo,* specifically from Lambda exonuclease (Exo) (Putney et al., 1981; Mosberg et al., 2012). Terminal PT linkages can easily be added to LETs during PCR with modified primers. However, these DNA modifications have generally shown insignificant stabilization of LETs in CFEs (Zhu et al., 2020), with the exception of one instance where two PT linkages were added immediately upstream of the σ^70^ promoter (Sun et al., 2013). Even still, these PT linkages only improved LET expression by about 36%. Internal PT linkages might be more effective in protecting dsDNA from Lambda Exo degradation *in vivo,* as suggested by increased recombination frequency (Mosberg et al., 2012). In addition, several other chemical modifications can be made to LETs via PCR primers that might reduce their susceptibility to degradation, including carbon spacers and 2′-O-methoxy-ethyl bases. However, to our knowledge, these modifications have yet to be tested in CFEs.

Different PCR-based techniques for template DNA production have also been explored for generating templates that provide the highest protein yield while still avoiding the lengthy cloning steps required to make plasmids. With appropriately designed primers, PCR amplicons can be purified and ligated to create circularized templates that give protein yields comparable to plasmid DNA without cloning and transformation (Wu et al., 2007). Furthermore, dilute samples of these recircularized PCR amplicons can be amplified overnight via rolling circle amplification and used directly in CFEs at more suitable concentrations (Dopp et al., 2019). These approaches maintain shorter production times, which is desirable for high-throughput testing, but have not gained much popularity in other applications. Also, their use has been restricted to only T7 expression elements.

Finally, designing the LET sequence in ways that specifically prolong the half-life of the messenger RNA (mRNA) transcripts can significantly improve LET-based production. Increased mRNA half-life is essential for LET yield since it allows for continued protein production even after the LET has been compromised. Use of extracts from strains such as BL21 Star—which has reduced RNase activity due to a mutation in the RNase E gene—is one way to accomplish this. Additionally, the inclusion of various 3′-tail mRNA secondary structures can improve protein yield from LETs, with poly(G) tails and T7 terminator sequences increasing yield by nearly 2-fold and 3-fold, respectively (Ahn et al., 2005).

### Reduced Culture Temperatures

It is worth noting that simple modifications to the lysate preparation protocol can sometimes improve LET protein yield, a particularly desirable approach based on its simplicity. It has previously been reported that *E. coli* cultivation temperature can have a significant influence on the exonuclease activity of the lysate. Reducing culture temperature to 30°C was shown to approximately triple LET-driven protein yield compared to lysates cultured at 37°C, while plasmid-driven protein yield decreased slightly (Seki et al., 2008). However, others have reported no increased expression from LETs when using this method (Sun et al., 2013).

## Applications of Linear Expression Templates

While the discussion in *Approaches for Stabilizing Linear DNA in Cell-Free Expression Systems* often used protein yields as a metric to indicate the relative utility of methods to stabilize LETs, producing large quantities of protein is not the most compelling reason to use LETs. In fact, all of the approaches described above still led to protein yields from LETs below those from plasmids. When producing large amounts of protein is the goal, the use of plasmids is preferred not only due to the attendant improved expression, but also due to the relatively low cost of plasmid production. The most prominent benefits of LETs lie in their ability to accelerate synthetic biology prototyping and enable the expression of genes that would otherwise be toxic to produce *in vivo*.

### Circuit Prototyping

Rapid prototyping of genetic circuits has been significantly impacted by the use of LETs in CFEs. Genetic circuit design to date has largely been guided by trial-and-error testing, known in the field as the Design-Build-Test-Learn (DBTL) cycle. The length of the DBTL cycle can be substantially shortened by using LETs to avoid time-consuming cloning steps after every iteration. For example, LETs have been used to assemble feedforward loop circuits and quickly facilitate promoter optimization (Guo and Murray 2019). Other complex systems, such as synthetic genetic oscillators (Niederholtmeyer et al., 2015; Yelleswarapu et al., 2018) and four-piece genetic switches have also been be assembled with LETs (Sun et al., 2013). To quantify rates of transcription and translation in real time, fluorescent reporters can be used at the RNA and/or protein level and measured directly. One example of such an assay, PERSIA, uses LETs in CFEs to rapidly characterize several biological phenomena during the reaction (Wick et al., 2019). The activities of T7 promoter mutants have been efficiently characterized through the use of LETs by using the PCR products from an *in vitro* transcription screen directly in CFEs (Komura et al., 2018). Similarly, using a common reverse primer and unique forward primers, a LET library was quickly generated to test the spatial dependence of promoter and operator sequences at low cost in lysate-based CFEs (McManus et al., 2019).

### Engineering Synthetic Regulators

LETs can also expedite the lengthy screening time required for engineering synthetic regulators. RNA toehold switches are powerful riboregulators that have been used to detect nucleic acid sequences *in vivo* (Green et al., 2014) and in CFEs, including low-cost user-friendly diagnostics to detect pathogens (Pardee et al., 2016). A toehold-based sensing system consists of two pieces: the RNA toehold switch sequence responsible for translational regulation, and the *trans*-acting nucleic acid trigger sequence that binds to the switch and modulates its activity. The switch and/or the RNA trigger can be encoded on LETs and expressed in the cell-free reaction (Takahashi et al., 2018), which is particularly important because finding a switch with good performance characteristics (leakiness, limits of detection, etc.) requires screening multiple (and sometimes many) candidates. Also, triggers can be added directly as linear ssDNA oligos to quickly test for switch functionality for a given target, avoiding the encoding of RNA triggers on DNA templates entirely (Amalfitano et al., 2021).

While the above and many other applications of toehold switches have used (nuclease-free) PURE systems, LET-based toehold systems can also be used in crude cell lysate systems for a more cost-efficient way to screen switches. Using Chi DNA as a nuclease inhibitor, ssDNA trigger oligos were stabilized and detected with toehold switches in encapsulated CFEs (Garamella et al., 2019). RNA triggers encoded onto LETs were used in developing a novel crude lysate-based multiplexed diagnostic sensor (Zhang et al., 2021). Additionally, in the more general class of synthetic riboregulators, riboswitch development also suffers from inefficient trial-and-error design (Etzel and Mörl 2017) that can be addressed with LETs in CFEs. This approach has previously been used as a platform to model riboregulator kinetics and predict *in vivo* behavior (Senoussi et al., 2018).

### CRISPR-Cas Screening

Development and screening of CRISPR systems has also benefited from the rapid prototyping enabled by LETs in CFEs. CRISPR technologies harness the nuclease activities from what is essentially a bacterial “immune system” to execute functions ranging from precise and efficient DNA editing to indiscriminate cleavage of nucleic acid sequences. These functions have been used in a variety of applications from plant and animal genomic manipulation to therapeutics and diagnostics (Barrangou and Doudna 2016; Petri and Pattanayak 2018). However, the laborious process of screening protein function has imposed obstacles to how rapidly advances can be made. This bottleneck has been mitigated in part through the use of CFEs for rapid CRISPR-Cas characterization (Marshall et al., 2018). LETs amplified from genomic fragments containing multiple Cas proteins have been used in CFEs to discover CRISPR-Cas12a inhibitors (Watters et al., 2018). LETs encoding dozens of guide RNA switch candidates (guide RNAs coupled to RNA toehold switches) were used for rapid characterization in CFEs to develop a modular design scheme for regulating Cas12a activity with arbitrary RNA inducers (Collins et al., 2021). LETs have also been used to elucidate the protospacer-adjacent motif (PAM) sequences recognized by Cas nucleases (Maxwell et al., 2018). While LETs can be used to express a Cas protein as well as its guide RNA sequence, it is particularly advantageous to use LETs for Cas expression since certain Cas genes can be extremely difficult to clone into plasmid vectors containing promoters suitable for CFEs (Maxwell et al., 2018).

### Expression of Toxic Proteins

Beyond Cas, the expression of many metabolically taxing or toxic proteins has been facilitated by LETs. *In vivo*, some proteins can interfere with metabolic pathways or inhibit cell division, resulting in cell death. Since CFEs do not have these same requirements as cells, they are a promising system for expression of such proteins. However, use of plasmids as expression templates in CFEs still requires traditional plasmid cloning, which can be challenging for such toxic proteins. LETs eliminate this challenge, as they can be generated by PCR from a DNA template that is much easier to clone: a plasmid with a defective promoter. For example, the human G-protein coupled receptor (GPCR) has been expressed at cytotoxic levels in both lysate-based CFEs and recombinant protein systems using LETs (Haberstock et al., 2012). Antimicrobial peptides (AMPs), which act as a natural infection defense mechanism and are known to severely stunt bacterial growth, have been successfully expressed with CFEs using LETs (Dopp et al., 2019). Other classes of difficult-to-express proteins that have been expressed successfully off plasmids in CFEs include vaccine antigens (Welsh et al., 2012) and antibiotic efflux pumps (Wuu and Swartz 2008). Employing LETs in these applications could simplify template production or translation to other proteins in these classes.

### Other Applications

LETs also allow CFEs to produce larger protein libraries more easily. Protein microarrays are systems that screen the activity and interaction of many proteins in parallel, but their use is limited by persisting technical challenges including lengthy protein expression, purification, and immobilization steps that reduce the maximum diversity of proteins for array construction (Schinn et al., 2016). The same challenges do not arise with DNA arrays; thus, *in situ* protein arrays that can use nucleic acid arrays as a template for protein production are advantageous (He et al., 2008b). Two methods that use LETs and CFEs to create protein microarrays include the protein *in situ* array, PISA (He and Taussig 2001), and a DNA array to protein array approach, DAPA (He et al., 2008a). Both methods involve parallel *in situ* protein expression and surface capture using purification tags that can be easily added to LETs with PCR. With PISA, 35 fg of unpurified LET was sufficient for detectable production of GFP in sub-nL volumes (Angenendt et al., 2006; Berrade et al., 2011).

LETs have also been shown to be functional in various non-standard reaction environments for CFEs. Using Chi DNA as a nuclease inhibitor, LETs have been used in polymer microgels to synthesize functional malonyl-CoA synthetase (MatB) (Köhler et al., 2020). LETs have also been spotted on glass slides and aligned to microfluidic devices enclosing each LET into a reaction chamber (Gerber et al., 2009). Microfluidic devices have also been used to encapsulate LET-containing CFEs (with GamS inhibitor) into agarose hydrogel beads, which then withstood lyophilization, storage, and rehydration into a functional sensor for *P. aeruginosa* quorum-sensing molecules (Seto 2019). Moreover, with sufficient protection, LETs can be used in continuous reaction conditions as well. While batch operation is the simplest mode for CFEs, depletion and degradation of reagents can limit long-term productivity. One notable example of a continuous format to address this issue was the use of a multilayer microfluidic device engineered to conduct extended cell-free reactions, where LETs were used to create a screening process that was entirely *in vitro* (Niederholtmeyer et al., 2013).

## The Future of Linear DNA in Cell-Free Expression Systems

Advances in nuclease inhibition strategies to facilitate LET-based expression have made the applications described above, and many others, possible. While existing nuclease inhibition methods are sufficient for certain applications, there is still both room and a critical need for improvement. Further enhancement of linear DNA stability and protein yield from LETs in CFEs can drive innovation of novel synthetic biology research tools and broaden the scope of viable applications of CFEs.

### Improvements in Linear Expression Template-Based Expression

One insufficiently studied aspect of CFEs is the interplay between promoter strength and template DNA structure. Since a common goal for using LETs in CFEs is maximizing protein yield, and since a common concern with using LETs is diminished expression, most scientists designing LETs choose to use strong promoters. As a result, there is little literature characterization of the expression levels from different promoters from LETs. However, use of diverse promoters is often an integral part of engineering genetic circuits and cell-free technologies, meaning that an accurate characterization of promoter strength in LETs is important. Multiple investigations of synthetic σ^70^ constitutive promoters on LETs showed that the relative strengths seen in CFEs have no correlation to the strengths seen when using the same promoters on plasmids in CFEs or *in vivo* (Chappell et al., 2013; Sun et al., 2013).

One hypothesis is that this discrepancy is due to the relationship between transcriptional rate and DNA supercoiling. This is supported by experiments that show stronger correlations of different ribosome binding site (RBS) strengths between linear and plasmid templates, as RBS sequence and resulting mRNA structure are independent of DNA template conformation (Chappell et al., 2013). Others have also explored the effect of supercoiled linear DNA on transcription by testing two different genes in convergent, divergent, and tandem orientations on a single LET (Yeung et al., 2017). Transcription from each configuration was significantly different, most likely because less supercoiling occurs at free DNA ends compared to the center. Recently, a study that focused on the impact of LET length on performance in CFEs verified via atomic force microscopy (AFM) a significant increase in DNA supercoiling as the LET length increased (Nishio et al., 2021). This study also showed that increasing the LET length from 2.8 to 25.7 kbp improved cell-free expression approximately 3-fold, which could be attributed to the positive influence of DNA supercoiling. The benefits of extending LET length have been previously reported while supplementing with GamS, albeit on a much smaller scale, where a 2.4-fold increase in LET expression was observed after the addition of just five bp on each end (Sun et al., 2013). Nonetheless, DNA supercoiling, the significance of gene orientation, and LET length should be taken into consideration for optimal design of future LET-based technologies.

It is also important to identify whether linear DNA stabilization techniques are generalizable across lysates from different species of bacteria. *E. coli* extracts make up the majority of CFEs because their lysate is the simplest to prepare and provides high protein yields, and because *E. coli* is otherwise a workhorse model organism. However, *E. coli* extracts are not practical for every application, as they cannot support some functionalities easily (e.g., glycosylation). As a result, the use of other chassis organisms as lysate sources is growing, and thus so will the demand for expression from LETs in CFEs made from those lysates. Some stabilization techniques that are shown to be most effective in *E. coli* systems (e.g., GamS) are practically ineffective in other CFEs such as *V. natriegens* (Wiegand et al., 2019). This is perhaps to be expected, as different organisms can possess different nucleases. One example of a nuclease inhibition approach with potential to be effective in CFEs of different species would be the small molecule RecBCD inhibitors mentioned in *Nuclease Inhibition*, as they have been shown to also inhibit AddAB (a helicase/nuclease native to many bacteria) (Amundsen et al., 2012). Interestingly, Chi DNA sites have also been identified in other bacteria, such as *B. subtilis* and *L. lactis*, indicating that Chi DNA oligos could potentially be useful in extracts made from these bacteria (Chedin and Kowalczykowski 2002; Marshall et al., 2017). Chi DNA inhibition has recently been tested in *B. subtilis* CFEs, and only improved LET yield about 2-fold (Yim et al., 2020). At any rate, identifying which techniques are generalizable—and for those that are not generalizable, identifying alternatives or replacements as needed—will be important in moving this aspect of CFEs forward.

However, it may in fact turn out that lysate-based CFEs from some non-model organisms could actually improve the efficiency of LET-based expression. Chinese hamster ovary (CHO) cell extracts have been shown to allow for sufficient LET functionality to be used for production of “difficult-to-express” proteins (Thoring et al., 2016). Wheat germ CFEs are known to possess undetectable nuclease activity compared to other cell-free systems (Endo and Sawasaki 2003). Surprisingly, in yeast CFEs, expression from LETs has been shown to yield 40–60% more protein compared to a plasmid DNA template (Gan and Jewett 2014). These LETs included a Ω leader sequence for initiation, a T7 promoter, and a 3’ poly(A)_50_ tail. Lysates from both *S. frugiperda* (Sachse et al., 2013), and *P. putida* (H. Wang et al., 2018) allow efficient expression from LETs*,* with the latter allowing for LET-based expression at about 70% of plasmid-based yields in the absence of any nuclease inhibitors or DNA protection. However, the economic viability for any downstream applications of CFEs based on these other chassis organisms remains to be seen.

Simultaneous inhibition of multiple nucleases could have a substantial impact on linear DNA stability in CFEs. The majority of approaches to nuclease inhibition have focused on RecBCD as the primary DNA nuclease, but significant activity from other nucleases has been recognized yet typically overlooked (Sun et al., 2013). For example, ExoVII has been identified as a key dsDNA and ssDNA nuclease; its removal has improved the preservation of mutations on dsDNA termini *in vivo* (Mosberg et al., 2012). Fortunately, some of the widely used nuclease inhibitors can inhibit more than one nuclease. For example, GamS can also inhibit SbcCD, another endo/exonuclease in *E. coli* (Kulkarni and Stahl 1989). Other significant native *E. coli* nucleases include RecJ, ExoI, and ExoX (Mosberg et al., 2012). Generalized inhibition methods would further improve linear DNA stability and help bring LET-based gene expression closer to plasmid yields.

### Linear Expression Templates to Avoid Confounding Effects in Cell-Free Expression Systems

LETs also have the potential to solve a unique phenomenon sometimes seen in CFEs but infrequently reported. Our group has observed multiple instances where the addition of plasmids expressing unrelated proteins, or even with no gene insert (“empty vectors”), seems to increase protein production from a separate reporter plasmid (Miguez et al., 2021). This is perhaps counterintuitive, as one might expect transcription or translation to be limited by competition for resources, such that adding more plasmids would increase competition for expression resources and thus likely decrease expression from the original plasmid. We hypothesize that this effect could be caused (in certain expression regimes) by RNase competition. Expression from additional plasmids added to the system would increase the total concentration of RNA, which would increase “competition” for RNases and thus in turn yield increased half-life of mRNA transcripts. Even empty vectors contribute to increasing the total RNA concentration in the reaction due to expression from their selectable marker cassettes. Since LETs encode only the gene of interest and no selectable markers, different LETs could be added for each gene to be expressed without the confounding effects typically caused by plasmids.

### Aptamers as Sensing Tools in Cell-Free Expression Systems

In synthetic biology, the engineering of novel genetic circuits is limited most significantly by the characteristics and diversity of extant cellular machinery (Purnick and Weiss 2009). Specifically, in the development of biosensors using CFEs, limitations on the “biological parts” available in nature has led to increasing demand for *de novo* regulators and synthetic circuit elements with low crosstalk and high sensitivity (Green et al., 2014). The most prominent examples of success in this space allow for rapid and specific detection of arbitrary nucleic acid sequences, which is useful in developing sensors for pathogens and other microbes. Detection of small molecules, however, has been more challenging because there are no *de novo*-designable regulators to facilitate detection of arbitrary small molecules (Silverman et al., 2020). There is thus a critical need for analogous technologies that can be used in synthetic circuits to detect classes of molecules beyond nucleic acid sequences, including small molecules, proteins, and ions.

Aptamers could potentially fill this need. Aptamers are short oligonucleotides that bind to a specific target with high affinity. DNA aptamers are stable at room temperature, can be synthesized at low cost with negligible batch-to-batch variability, and can withstand lyophilization (Zhang et al., 2019). They have previously been used as the basis for robust sensors for the detection of all types of molecular targets. Most importantly, aptamers are evolved *in vitro*, which means new aptamers can be created for virtually any analyte.

Despite all of the strengths of DNA aptamers, there are surprisingly few examples of their use in CFEs, likely due to the instability of linear DNA in bacterial extracts. As discussed in *Approaches for Stabilizing Linear DNA in Cell-Free Expression Systems*, even the best methods for stabilizing LETs still allow significant degradation. DNA aptamers, unlike LETs, are mostly ssDNA oligos, which likely increases their susceptibility to enzymatic degradation in *E. coli* since there are more native exonucleases that act on ssDNA specifically than there are that act solely on dsDNA (Lovett 2011). Also, aptamers are significantly shorter in length than LETs: LETs include entire coding sequences and other transcription elements, while evolved aptamers are normally 20–60 nucleotides long (Lakhin et al., 2013). Because we know that nucleases can cleave DNA at rates as high as 500 bp/s (Spies et al., 2007) and that extending LETs results in improved protein yield (Sun et al., 2013), it is likely that the short lengths of aptamers are a major contributor to their instability. This seems reasonable because on a mass basis, aptamers have more free ends than LETs for exonucleases to bind to, and on a molar basis they may have a higher density of critical sequence without which they cannot function.

**FIGURE 3:**
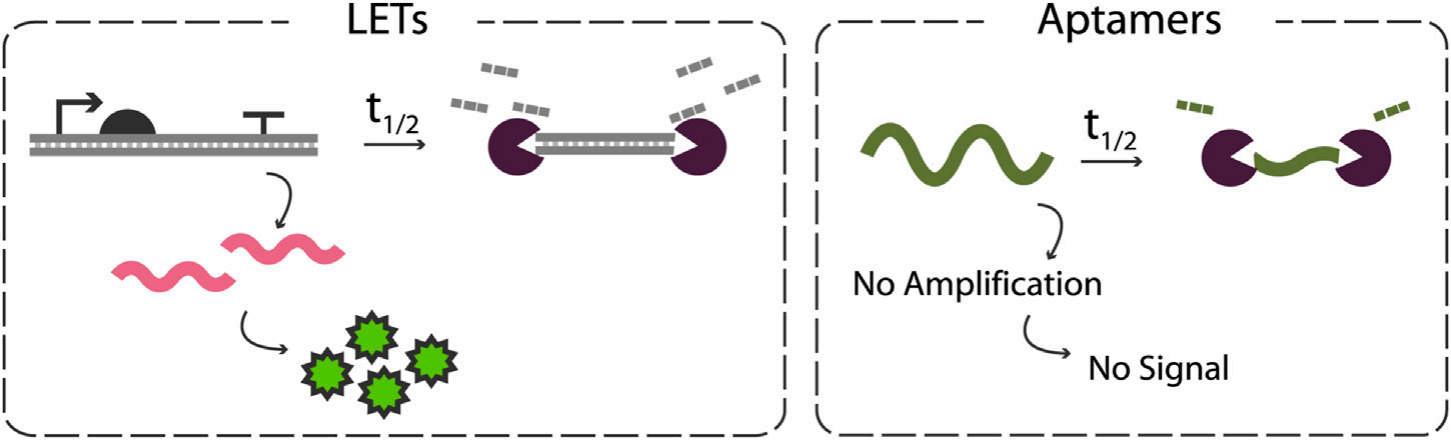
Comparison of the impacts of degradation on LETs vs DNA aptamers in CFEs. LETs benefit from downstream amplification steps, leading to continued protein synthesis even after complete LET degradation. Aptamers, however, must be present and intact to execute their function as they do not serve as templates for amplification.

Another characteristic that sets aptamers apart from LETs is that aptamers do not undergo downstream amplification steps like LETs do (Figure 3). LETs produce numerous mRNA transcripts which are then amplified into protein. These transcripts remain viable templates for protein translation even after the LET has been degraded, and the proteins themselves may also have some function that serves as an amplification of signal in certain applications. Aptamers, however, are not templates for amplification and thus leave no residual function after enzymatic degradation. This means that for aptamers to be used successfully in lysate-based CFEs, nuclease inhibition strategies must be strong enough to prevent aptamer degradation for the entirety of the reaction.

In a few cases, these obstacles have been overcome or circumvented sufficiently to allow DNA aptamers to be successfully used in CFEs. For example, DNA aptamers integrated into plasmids have successfully allowed transcriptional regulation in CFEs (Iyer and Doktycz 2014; Wang et al., 2017). This confirms that the environment of CFEs (in these examples, a commercially available extract) can accommodate proper aptamer secondary structure formation to allow for both target analyte binding and subsequent conformational changes. Integrating the aptamer onto a circular template was done to mitigate exonuclease activity that would otherwise degrade the aptamer. To that end, these reports do not indicate the use of any additional nuclease inhibitors in their experiments.

However, the integration of aptamers onto circular templates can be significantly more labor-intensive and challenging than most of the LET stabilization techniques described above. Since aptamers are single-stranded, incorporation into a circular template typically requires generation of a ssDNA circular template by affinity purification of biotin-labeled ssDNA produced via PCR (Mitchell and Merril 1989). This approach has the advantage of *in vitro* production but often produces insufficient yields for downstream applications. An alternative method that can produce a higher yield of ssDNA entails *in vivo* amplification in *E. coli* with phagemids, which are plasmids that possess both bacteriophage and plasmid properties and thus have all the viral components necessary to enable ssDNA replication (Kuczek et al., 1998; Zhou et al., 2009). However, this approach is even more complex than the *in vitro* strategy. Regardless of the synthesis strategy, constraining DNA aptamers to circular templates limits the design space and could compromise the functionality of some aptamers. Given all of these considerations, the development of simpler, more effective, and more generalizable methods to enable their stability are a critical need to unleash the potential of DNA aptamers in CFEs.

## Conclusion

The advances made in nuclease inhibition (specifically of RecBCD) have improved linear DNA stability in lysate-based CFEs sufficiently to allow feasible LET-based production of proteins. The accessibility and ease of implementation of these methods has supported their effective use in driving innovative research in genetic circuit design, efficient screening of *de novo* riboregulators, rapid screening of CRISPR-Cas system parts, and more. However, continued progress is necessary, because while protein yields from LETs have improved significantly, this is not a direct measurement of LET half-life and obscures the difficulties in using other types of linear DNA constructs in CFEs. Keeping linear DNA stable long enough to function on its own, rather than as a template for amplification, is an important challenge to tackle. Some of the potentially most promising applications of linear DNA in CFEs remain infeasible due to nuclease-based degradation, meaning that more effective methods to extend linear DNA half-life could have a dramatic, enabling impact on a myriad of biotechnology applications.
