# Corrigendum: Effective use of linear DNA in cell-free expression systems

**DOI:** 10.3389/fbioe.2022.979285

**Published:** 2022-08-08

**Authors:** Megan A. McSweeney, Mark P. Styczynski

**Affiliations:** Georgia Institute of Technology, School of Chemical & Biomolecular Engineering, Atlanta, GA, United States

**Keywords:** cell-free expression, linear expression template, nuclease inhibition, genetic circuits, rapid prototyping, DNA aptamers

In the original article, there was an error in the **Funding** statement. The support of the National Science Foundation was omitted. The correct Funding statement appears below.

Funding

“The authors thank the National Institutes of Health (R01-EB022592) and the National Science Foundation (CCF-2007807) for funding support.”

The authors apologize for this error and state that this does not change the scientific conclusions of the article in any way. The original article has been updated.

